# A phase 2 study of bortezomib, cyclophosphamide, pegylated liposomal doxorubicin and dexamethasone for newly diagnosed multiple myeloma

**DOI:** 10.1038/bcj.2016.31

**Published:** 2016-05-13

**Authors:** P S Becker, T A Gooley, D J Green, N Burwick, T Y Kim, K Kojouri, Y Inoue, D J Moore, E Nelli, T Dennie, W I Bensinger

**Affiliations:** 1 Divisions of Hematology and Medical Oncology, University of Washington, Seattle, WA, USA; 2 Clinical Research Division, Fred Hutchinson Cancer Research Center, Seattle, WA, USA; 3 Seattle Cancer Care Alliance, Seattle, WA, USA; 4 Skagit Valley Hospital Regional Cancer Care Center, Mount Vernon, WA, USA; 5 Providence Regional Cancer Partnership, Everett, WA, USA; 6 SCCA at Evergreen Health, Kirkland, WA, USA; 7 Hematology Oncology Associates, Medford, OR, USA; 8 MultiCare Health System, Tacoma, WA, USA; 9 Swedish Cancer Institute, Seattle, WA, USA

The goal of initial treatment for transplant eligible patients with multiple myeloma (MM) is to achieve the deepest possible response in an effort to attain prolonged event-free survival after transplant. There has been an excellent response to the three-drug regimens of agents approved for upfront use (Table 1), including bortezomib/IMiD (thalidomide or lenalidomide) dexamethasone (VTD or VRD) and bortezomib/cyclophosphamide/dexamethasone (VCD). We had previously treated patients with three cycles each of two sequential three-drug regimens, VCD, then VTD, and reported an overall response rate of 92%, with a CR rate of 26%.^1^ Another three-drug regimen, liposomal doxorubicin/bortezomib/dexamethasone (DVD) also resulted in a good overall response rate of 71.5%⩾PR, and 20% CR in previously untreated patients.^2^ Our objective in developing the bortezomib, cyclophosphamide, pegylated liposomal doxorubicin and dexamethasone regimen was to improve the depth of response and overall response rate compared to three-drug regimens. In addition, the study was designed to improve ease of administration by use of weekly dosing rather than the typical twice weekly dosing (that is, days 1, 4, 8 and 11) of bortezomib (the standard dosing at study inception). The efficacy of this four-drug regimen was examined in newly diagnosed, transplant eligible patients, with a secondary objective of evaluating rates of successful stem cell mobilization and survival after transplant.

This study was conducted with approval of the University of Washington-Fred Hutchinson Research Center Cancer Consortium Institutional Review Board, and the Institutional Review Boards of the Seattle Cancer Care Alliance Network sites. Written informed consent was obtained from all patients. The trial was registered as NCT00849251 on www.clinicaltrials.gov.

This study was comprised of two cohorts. After a pilot phase to assess tolerability in the relapsed setting (cohort 1), then newly diagnosed patients were enrolled (cohort 2). Relapsed, refractory patients with multiple myeloma who had failed at least one prior regimen, not including dexamethasone alone, were eligible to enroll in cohort 1. Newly diagnosed patients with previously untreated MM other than prior dexamethasone that did not exceed a total dose of 320 mg were eligible for cohort 2. Patients who were 18 years and older with quantifiable monoclonal protein or light chain identified by serum protein electrophoresis, urine protein electrophoresis or serum-free light-chain assay were enrolled. Eastern Cooperative Oncology Group performance status was 0–2. Patients were required to have adequate blood counts, renal, hepatic and cardiac function. Patients with uncontrolled infection were excluded, as were patients with grade 2 or higher neuropathy, prior cumulative dose of 400 mg/m^2^ of doxorubicin or equivalent, patients with hypersensitivity to boron or bortezomib, those who were pregnant or lactating, patients with other cancers with limited exceptions, or patients who had undergone prior autologous or allogeneic transplant.

The regimen consisted of bortezomib, 1.6 mg/m^2^ IV, cyclophosphamide 300 mg/m^2^ IV, and dexamethasone, 40 mg po or IV, on days 1, 8 and 15, and liposomal doxorubicin 30 mg/m^2^ IV on day 8 of a 28 day cycle. Four cycles were intended to be completed before proceeding to autologous stem cell transplant (ASCT).

The primary objective was to determine efficacy of the BCDD regimen in newly diagnosed patients with MM, evaluated according to the criteria of the International Myeloma Workshop Consensus Panel.^3^ The secondary objectives were to determine (1) the toxicity of BCDD and (2) outcomes after ASCT.

After five patients with relapsed disease (cohort 1) were treated at Seattle Cancer Care Alliance without incident, the Institutional Review Board approved initiation of enrollment of cohort 2, the newly diagnosed patients. Ten cohort 2 patients were treated at Seattle Cancer Care Alliance Network community affiliates. Enrollment goals were ultimately modified due to the lack of availability of pegylated liposomal doxorubicin (Doxil)for several months, leading to a total enrollment (Supplementary Data) of 31 patients (both newly diagnosed and relapsed) of the 45 planned. For the five relapsed patients who received 2–4 cycles of treatment, the responses were one very good partial response (VGPR) (nCR), one partial response (PR), two minimal response and two stable disease. For the 20 patients with newly diagnosed MM who completed four cycles of treatment, there were two complete responses (CRs), six VGPRs (of which one was nCR), 10 PRs, and two stable disease for an overall (CR+VGPR+PR) response rate of 90%. Five patients did not complete four cycles of therapy, one due to massive pulmonary embolism, one because of need for radiation for intractable back pain during cycle 2 despite marked serological response and three with plateau in response, and decision to change therapy.

After treatment, 21 patients proceeded to successful mobilization and collection of peripheral blood stem cells, and autologous or tandem autologous (2) or tandem autologous then reduced intensity allogeneic stem cell transplant (8). Mobilization was with filgrastim (1), cyclophosphamide/dexamethasone (4), cyclophosphamide/etoposide/dexamethasone (CED) (4), bendamustine/etoposide/dexamethasone (BED) (5), and VRD-PACE (7) and VTD-PACE (1). One patient had two mobilization regimens, CED followed by BED. Eleven patients completed collection in one day, six in 2 days, three in 3 days and one in 4 days. For the recipients of reduced intensity allogeneic transplants, two had matched related sibling donors, and six had matched unrelated donors.

Out of the 25 patients who received BCDD as initial therapy, there have been five deaths to date, one due to massive pulmonary embolism on day 13 of the first cycle of treatment, without known history of hypercoagulable risk, one at 8 and one at 18 months of unknown cause, and one at 15 and one at 34 months of progressive disease, resulting in an estimated overall survival of 80% at 3 years from start of therapy (Figure 1a). For high- risk cytogenetics, the estimated overall survival at 3 years is 71.4%, and for standard risk, 83.3% (Figure 1b). For International Staging System^4^ stage I, the estimated overall survival at 3 years is 88.8%, stage II 82.8%, and International Staging System stage III 60% (Figure 1c). Median follow-up among the 20 survivors is 49 months (range 36–56 months). Median survival has not been reached for any of the groups or all patients.

One patient with a known central line associated deep venous thrombosis in the relapsed group did not exhibit progression of thrombosis off warfarin during therapy. One patient in the new diagnosis group sustained a massive pulmonary embolism resulting in death on day 13 of therapy. After enrollment of the first nine patients, an amendment was filed for subsequent patients to receive aspirin prophylaxis, or if at high risk by criteria proposed by Palumbo *et al.*^5^ for prophylaxis for MM patients on IMiDs, with low molecular weight heparin or warfarin.

The grade 3 adverse events included hand/foot syndrome (2), infection without neutropenia (2), and gastrointestinal hemorrhage due to Mallory–Weiss tear (1), diarrhea (1), weight loss (1), anemia (1), mucositis (1) and chronic obstructive pulmonary disease exacerbation (1).

After ASCT, 8 out of 21 (38%) patients had achieved complete response, five had achieved VGPR (24%), six had achieved PR (29%), for an overall response rate of 90%, based on day +80 re-staging. Three of the 21 patients have died after transplant, one at 2 months after first ASCT from unknown cause, one at 10 months after ASCT from progressive disease and one who underwent autologous then reduced intensity allogeneic transplant who died of progressive disease at 45 months from the autologous/43 months from the allogeneic transplant. Median follow-up after first ASCT among the 18 survivors is 45 months (range 37–54 months).

A dose escalation study employing a regimen comprised of the same 4 drugs (CVDD),^6^ tested two dose levels of bortezomib, 1.0 or 1.3 mg/m^2^ per dose, days 1, 4, 8 and 11, and increasing doses of cyclophosphamide as a single dose on day 1 of each 21 day cycle, 250, 500 or 750 mg/m^2^, with liposomal doxorubicin 30 mg/m^2^ on day 4 and dexamethasone 20 mg per dose on days 1, 2, 4, 5, 8, 9, 11 and 12. There was an comparable overall response rate of 93% but greater toxicity, as 59% of patients in the other study required dose reduction, 56% for neuropathy and 46% for hand-foot syndrome, as compared with no need for dose reduction in our study, with 0% neuropathy and 6% hand-foot syndrome of grade 3 toxicity. Other four-drug combinations have been studied, including lenalidomide, bortezomib, liposomal doxorubicin and dexamethasone (RVDD),^7^ and bortezomib, dexamethasone, cyclophosphamide and lenalidomide (VDCR),^8^ that each achieve high response rates, 96%⩾PR, 35% CR+ nCR for RVDD and 88%⩾PR, 5% CR for VDCR. The rate of VGPR or CR seen here may not be as high as some other studies (Table 1), as we limited the number of cycles to 4, and for the VRD regimen, that there was an increase in CR rate from 6 to 39% during cycles 5–8.^9^

In summary, the four-drug BCDD regimen was well tolerated, and was convenient for patients, as it was administered weekly for 3 weeks out of the 4 week cycles. It was easily administered in outpatient offices in the community. The induction regimen successfully prepared patients for transplant, with preservation of ability to mobilize and collect peripheral blood stem cells. There is excellent estimated overall survival, 80% at 3 years. The overall response rate was 90%. The response rate and overall survival after BCDD and current consistent availability of pegylated liposomal doxorubicin support a future direct comparison of BCDD to other drug regimens.

## Figures and Tables

**Figure 1:**
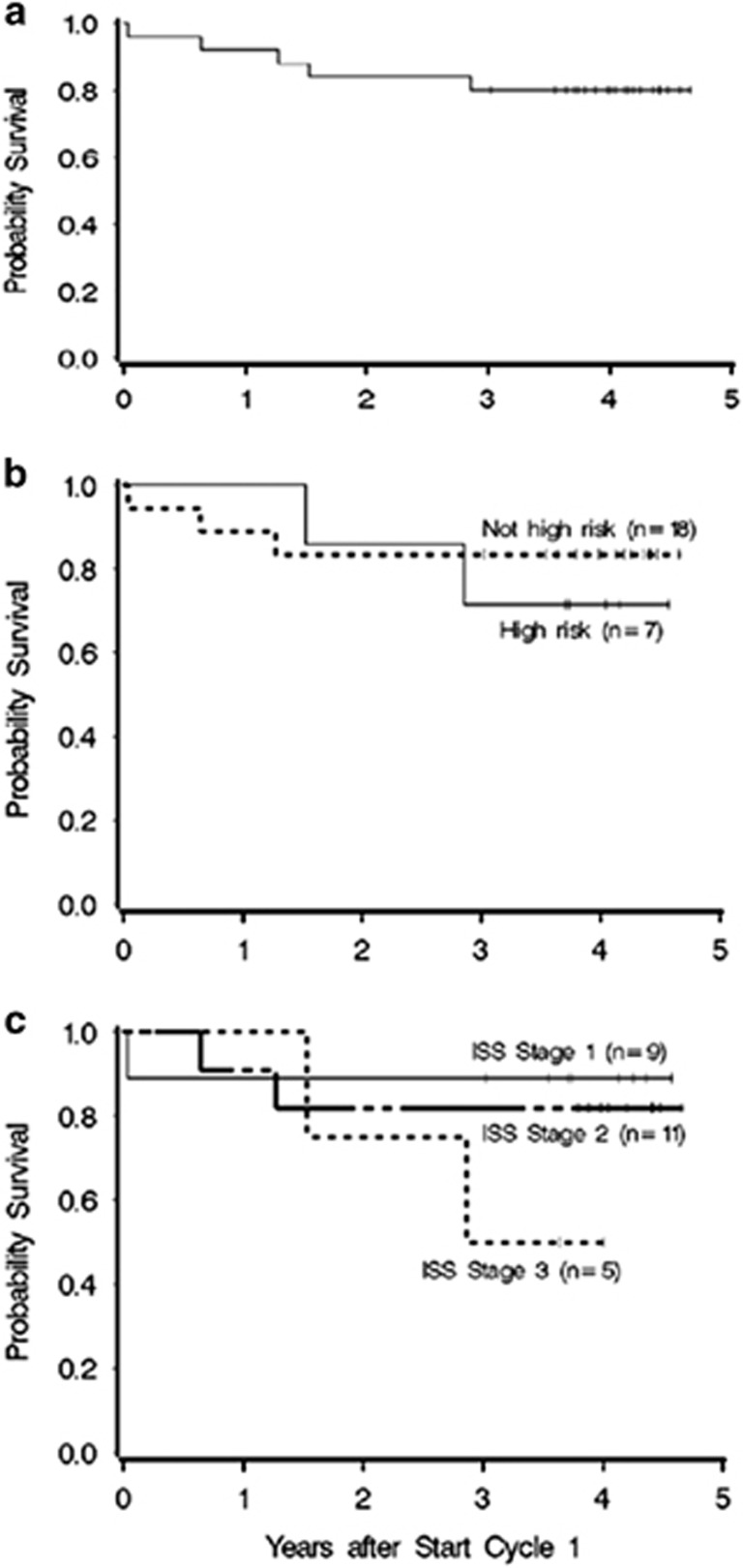
(**a**) Overall survival. The estimated overall survival at 3 years is 80%. (**b**) Survival by cytogenetic risk group. For high-risk cytogenetics [t(4;14), t(14;16), del 17p or del 13 (del 13 by karyotype)], the estimated overall survival (OS) at 3 years is 71.4%, and for the others, 83.3%. (**c**). Survival by ISS (International Staging System) Stage. For ISS stage I, the estimated OS at 3 years is 88.8%, stage II 82.8%, and ISS stage III 60%.

**Table 1:** Summary of responses to upfront three- and four-drug regimens with drugs that are approved for initial treatment

*Regimen*	*Response*		*Reference*
VTD	88% ⩾VGPR	13% CR	^10^
VRD	67% ⩾VGPR, 11% after four cycles	39% CR+nCR, 6% after four cycles	^9^
VDR	32% ⩾VGPR	7% CR	^8^
VDC	13% ⩾VGPR	3% CR	^8^
DVD	29%⩾VGPR	20% CR	^2^
CyBorD	60–65%⩾VGPR	41% CR+nCR	^11, 12^
VCD then VTD	57% ⩾VGPR	26% CR	^1^
CRD	30%⩾VGPR	2% CR+nCR	^12^
CRd	47%⩾VGPR, 30% after four cycles	2% CR	^13^
RVDD	57% ⩾VGPR (four cycles)	35% CR+nCR	^7^
VDCR	58% ⩾VGPR	5% CR	^8^
CVDD	88%⩾VGPR high risk and 63% std risk	26% CR	^6^
BCDD	40% ⩾VGPR (four cycles)	10% CR (15% CR+nCR) (four cycles)	Current study

Abbreviations: C, cyclosphamide; CR, complete response; D, dexamethasone (or pegylated liposomal doxorubicin in DVD); R, lenalidomide; T, thalidomide; V or B or Bor, bortezomib; VGPR, very good partial response.

## References

[bib1] Bensinger WI, Jagannath S, Vescio R, Carnacho E, Wolf J, Irwin D et al. Phase 2 study of two sequential three-drug combinations containing bortezomib, cyclophosphamide and dexamethasone, followed by bortezomib, thalidomide and dexamethasone as frontline therapy for multiple myeloma. Br J Haematol 2010; 148: 562–568. 19919652 10.1111/j.1365-2141.2009.07981.x

[bib2] Berenson JR, Yellin O, Chen CS, Patel R, Bessudo A, Boccia RV et al. A modified regimen of pegylated liposomal doxorubicin, bortezomib, and dexamethasone (DVD) is effective and well tolerated for previously untreated multiple myeloma patients. Br J Haematol 2011; 155: 580–587. 21950583 10.1111/j.1365-2141.2011.08884.x

[bib3] Rajkumar SV, Harousseau J-L, Durie B, Anderson KC, Dimopoulos M, Kyle R et al. Consensus recommendations for the uniform reporting of clinical trials: report of the International Myeloma Workshop Consensus Panel. Blood 2011; 117: 4691–4695. 21292775 10.1182/blood-2010-10-299487 PMC3710442

[bib4] Greipp PR, San Miguel J, Durie BG, Crowley JJ, Barlogie B, Bladé J et al. International staging system for multiple myeloma. J Clin Oncol 2005; 23: 35412–35420. 10.1200/JCO.2005.04.242 15809451

[bib5] Palumbo A, Rajkumar SV, Dimopoulos MA, Richardson PG, San Miguel J, Barlogie B et al. Prevention of thalidomide- and lenalidomide-associated thrombosis in myeloma. Leukemia 2008; 22: 414–423. 18094721 10.1038/sj.leu.2405062

[bib6] Nishihori T, Baz R, Shain K, Kim J, Ochoa-Bayona JL, Yue B et al. An open-label phase I/II study of cyclophosphamide, bortezomib, pegylated liposomal doxorubicin, and dexamethasone in newly diagnosed myeloma. Eur J Haematol 2015; 95: 426–435. 25600676 10.1111/ejh.12509 PMC4508238

[bib7] Jakubowiak AJ, Griffith KA, Reece DE, Hofmeister CC, Lonial S, Zimmerman TM et al. Lenalidomide, bortezomib, pegylated liposomal doxorubicin, and dexamethasone in newly diagnosed multiple myeloma: a phase 1/2 Multiple Myeloma Research Consortium trial. Blood 2011; 118: 535–543. 21596852 10.1182/blood-2011-02-334755 PMC3142898

[bib8] Kumar S, Flinn I, Richardson PG, Hari P, Callander N, Noga SJ et al. Randomized, multicenter, phase 2 study (EVOLUTION) of combinations of bortezomib, dexamethasone, cyclophosphamide, and lenalidomide in previously untreated multiple myeloma. Blood 2012; 119: 4375–4382. 22422823 10.1182/blood-2011-11-395749

[bib9] Richardson PG, Weller E, Lonial S, Jakubowiak AJ, Jagannath S, Raje NS et al. Lenalidomide, bortezomib, and dexamethasone combination therapy in patients with newly diagnosed multiple myeloma. Blood 2010; 116: 679–686. 20385792 10.1182/blood-2010-02-268862 PMC3324254

[bib10] Moreau P, Avet-Loiseau H, Facon T, Attal M, Tiab M, Hulin C et al. Bortezomib plus dexamethasone versus reduced-dose bortezomib, thalidomide plus dexamethasone as induction treatment before autologous stem cell transplantation in newly diagnosed multiple myeloma. Blood 2011; 118: 5752–5758. 21849487 10.1182/blood-2011-05-355081

[bib11] Reeder CB, Reece DE, Kukreti V, Chen C, Trudel S, Laumann K et al. Once- versus twice-weekly bortezomib induction therapy with CyBorD in newly diagnosed multiple myeloma. Blood 2010; 115: 3416–3417. 20413666 10.1182/blood-2010-02-271676

[bib12] Khan ML, Reeder CB, Kumar SK, Lacy MQ, Reece DE, Dispenzieri A et al. A comparison of lenalidomide/dexamethasone versus cyclophosphamide/lenalidomide/dexamethasone versus cyclophosphamide/bortezomib/dexamethasone in newly diagnosed multiple myeloma. Br J Haematol 2012; 156: 326–333. 22107129 10.1111/j.1365-2141.2011.08949.x

[bib13] Kumar SK, Lacy MQ, Hayman SR, Stewart K, Buadi FK, Allred J et al. Lenalidomide, cyclophosphamide and dexamethasone (CRd) for newly diagnosed multiple myeloma: results from a phase 2 trial. Am J Hematol 2011; 86: 640–645. 21630308 10.1002/ajh.22053 PMC3901994

