# Supplementary material for: A phase 2 study of bortezomib, cyclophosphamide, pegylated liposomal doxorubicin and dexamethasone for newly diagnosed multiple myeloma
Source: Blood Cancer J. 2016 May 13;6(5):e422–. doi: 10.1038/bcj.2016.31 (PMC4916300; doi:10.1038/bcj.2016.31)
Supplement: Supplementary Data [file bcj201631x1.doc]

**Supplementary Table 1.Clinical Features.**

| **Characteristic** | Number |
| --- | --- |
| **Age**  Median 59  Range 41-78 | Total=31 |
| **Gender**  Male  Female | 14  17 |
| **International Staging System**  Stage 1  Stage 2  Stage 3 | 10  16  5 |
| **High Risk Cytogenetics**  t(4;14)  del 17p  del 13 by karyotype | 6  3  2 |
| **Type of Monoclonal Immunoglobulin**  IgG kappa  IgG lambda  IgA kappa  IgA lambda  IgD lambda  Kappa only | 13  9  2  4  1  1 |

**Supplementary Table 2. Courses Completed.** C=cycle number, D=day number

| **Number Patients** | **Newly Diagnosed** | **Relapsed** | **Cycles** |
| --- | --- | --- | --- |
| 4 |  | X | 4 |
| 1 |  | X | 2 |
| 20 | X |  | 4 |
| 1 | X |  | C1D13 |
| 1 | X |  | C1C2D1 |
| 2 | X |  | 2 |
| 1 | X |  | 3 |
